# Fetal Health State Detection Using Interval Type-2 Fuzzy Neural Networks

**DOI:** 10.3390/diagnostics13101690

**Published:** 2023-05-10

**Authors:** Rahib Abiyev, John Bush Idoko, Hamit Altıparmak, Murat Tüzünkan

**Affiliations:** 1 Applied Artificial Intelligence Research Centre, Department of Computer Engineering, Near East University, Nicosia 99138, Turkey; 2 Department of Computer Engineering, Near East University, Nicosia 99138, Turkey; 3 Applied Artificial Intelligence Research Centre, Near East University, Nicosia 99138, Turkey

**Keywords:** fetal health status, type-2 fuzzy neural system, diagnose, fetal healthy rate

## Abstract

Diagnosis of fetal health is a difficult process that depends on various input factors. Depending on the values or the interval of values of these input symptoms, the detection of fetal health status is implemented. Sometimes it is difficult to determine the exact values of the intervals for diagnosing the diseases and there may always be disagreement between the expert doctors. As a result, the diagnosis of diseases is often carried out in uncertain conditions and can sometimes cause undesirable errors. Therefore, the vague nature of diseases and incomplete patient data can lead to uncertain decisions. One of the effective approaches to solve such kind of problem is the use of fuzzy logic in the construction of the diagnostic system. This paper proposes a type-2 fuzzy neural system (T2-FNN) for the detection of fetal health status. The structure and design algorithms of the T2-FNN system are presented. Cardiotocography, which provides information about the fetal heart rate and uterine contractions, is employed for monitoring fetal status. Using measured statistical data, the design of the system is implemented. Comparisons of various models are presented to prove the effectiveness of the proposed system. The system can be utilized in clinical information systems to obtain valuable information about fetal health status.

## 1. Introduction

Fetal distress is an abnormal phenomenon that results in low or high fetal heart rate (FHR) which refers to the number of heart beat rates per minute (BPM). Cardiotocogram (CTG) is the primary method for fetal status detection that is most frequently employed in clinical routine examinations. Fetal heart rate and uterine contractions (UC) are the two main physiological signs used in prenatal monitoring of CTG. FHR is affected by fetal distress that leads to low and or high FHR abnormal phenomena. This information is used for the early detection of a pathological state. CTG data can be used to classify the pathological state of the fetus relative to normal indicating the healthy state. The hypoxic fetus is very sensitive and vulnerable to temporary impairment or death during delivery. Inappropriate treatment and misdiagnosis of FHR can be more than half of the reason behind the death caused. The number of neonatal seizures is decreased with continuous CTG during delivery. The design of a system for the early detection of a fetus provides clinicians with important pathological and physiological information about the fetus in pregnant women, effectively preventing premature birth.

One of the most difficult and intricate procedures in medicine is tracking fetal growth during pregnancy. Even when preventative steps are implemented, approximately 810 pregnant women still pass away every day, according to the World Health Organization (WHO) [1]. In affluent nations, the maternal mortality ratio (MMR) is noticeably low, while it is high in poor nations. Preeclampsia, inadequate monitoring of the mother’s health and the state of the unborn child, and gestational diabetes are common problems that contribute to high MMR [2]. With the right medical care, MMR can be mitigated and even avoided. Fetal monitoring is a routine practice carried out in the third trimester. Monitoring of the fetus’ health is performed during pregnancy. The health of the mother has no bearing on fetal growth. Cardiotocography is used to continuously measure the health and growth rate of the fetus to prevent any issues. The goal of cardiotocography is to monitor the fetus’ heartbeat and gauge the mother’s uterine contractions simultaneously. When the fetus’ growth is fully coordinated with heart rate during the third trimester, this process would be carried out. This technique is used by medical professionals for early fetal status detection and to lower fetal mortality because it is thought to be simple and cost effective. Cardiotocographic (CTG) results show the mother’s uterine contractions as well as the fetus’s heart rate, accelerations, decelerations, and other intricate measurements [3]. The normal, suspected, and abnormal stages of the fetus can be distinguished using different machine learning methods. The outcomes demonstrate that the machine learning (ML) technique creates a framework that is frequently employed for the automated system In the analysis of early fetal health [4].

Diagnosis of fetal health is a difficult process that depends on various input factors. Depending on the values or the interval of values of these symptoms, the diagnosis of fetal healthy status is implemented. Sometimes it is difficult to determine the exact values of the intervals of the input symptoms that affect the output of diagnosis. The doctors often partition the whole interval value of symptoms and analyze each of the partitioned intervals and make a decision on the level of health conditions of the patient. Often, these intervals can be different for each patient, and carry uncertainties. At the same time, different patients may react to the same diseases at different degrees. As a result, the diagnosis of diseases is often carried out in uncertain conditions and can sometimes cause undesirable errors. Therefore, the vague nature of diseases and incomplete patient data can lead to uncertain decisions. One of the effective approaches to solve such kind of problem is the use of fuzzy logic in the design of the diagnostic system. Fuzzy logic can adequately describe the uncertainty existing in input symptoms. Fuzzy logic systems using linguistic terms with their numerical approximation can describe uncertain knowledge [5]. Therefore, in this paper, fuzzy logic is employed for the detection of fetal health status.

## 2. Related Research Works

Due to stability and effectiveness, machine learning and deep learning approaches have recently been widely used in various medical imaging disciplines. These research studies include the classification of medical diseases, the segmentation of disorders and the detection and segmentation of anatomical images [6]. As a result, numerous sophisticated diagnostic methods for fetal status (FS) images were presented. For instance, in Ref. [7], the authors proposed a unique framework to use ultrasound images to identify prenatal abnormality. The abdominal region is first segmented using the framework’s U-Net architecture and Hough transformation, and then a multistage convolutional neural network (CNN) is created to extract the hidden characteristics of images of FS. It performed better than other CNN-based approaches, according to the experiment in Ref. [7]. To solve the issue of standard object detection and evaluation of fetal health using ultrasound images, Lin and colleagues [8] suggested a multitask CNN framework. To further lower the false detection rate, they added prior clinical and statistical knowledge to the system. This method’s detection speed is quite fast, and the performance it produces is promising when compared to cutting-edge techniques [8].

Medical professionals can reduce the MMR and high labor complications by using machine learning (ML) tools to aid in early decision-making in difficult situations such as diagnosis. Although classifying the fetal health states is a difficult task, the ML classification systems are perfect at handling it [9]. Random forest, neural networks, and SVM are a few of the common classification techniques [9]. The random forest classifier performs better and more accurately when dividing the stages of fetal health. Prenatal mortality can be lowered at random by monitoring the fetus even in the second trimester [10]. A critical early diagnostic decision has lately been made possible using artificial intelligence (AI) approaches. A comprehensive comparison between 15 machine learning approaches was conducted for healthy and unhealthy fetuses [11]. The features are taken from the recorded CTG signal. These efficiently assess massive real-time datasets to deliver improved performances and create a framework for other models to carry out classification [12]. CTG signals are used to directly measure a patient’s heart rate and provide reliable information and updates to medical professionals. The effectiveness of employing CTG for the fetal welfare during labor is discussed. The fetal heart rate and womb contractions are measured by CTG, which is also used to determine the frequency of the baby’s movements. As a result, CTG is essential for fetal assessment both before and during labor. In [13], an enhanced binary bat algorithm is used for the classification of fetal status. The research used feature extraction to improve the results. In Ref. [14], the authors used the Bagging ensemble machine learning (ML) algorithm for the classification of fetal heart rate signals. The authors obtained satisfactory results using bagging ensemble with a random forest algorithm. In Ref. [15], the authors used decision tree, SVM and Naïve Bayes algorithms on R-Studio tools for the classification of fetal health status. The decision tree has shown better performance than other ones. In Ref. [16], the authors deployed various ML algorithms for the prediction of fetal health from the CTG data. The authors obtained better results using random forest. In Ref. [17], the authors used ML techniques on CTG data for identifying high-risk fetuses. The models based on XGBoost, decision tree and random forest showed better high precision. In Ref. [18], the authors used ML techniques to predict fetal anomalies. Nine ML algorithms were tested using a clinical dataset of 96 pregnant women. In Ref. [19], the authors used new CTG dataset for the extraction of features. The classification of the selected features was implemented using a synthetic minority oversampling technique and the nearest mean classifier with Adaboost. In Ref. [20], the authors presented an approach for the evaluation of missing data in fetal heart rate datasets. Two iterative steps using an empirical dictionary and the construction of the dictionary using the updated values were presented.

As mentioned in the above section, the diagnosis of fetal health depends on various input factors. The doctors often make a decision on the level of health conditions of the patient using the interval value of these input symptoms. Often, these intervals carry uncertainties. Fuzzy logic can adequately describe these uncertainties existing in input symptoms. There exist several research papers related to the diagnosis of diseases using fuzzy logic. In Ref. [21], the authors designed a Mamdani-type fuzzy system for diagnosing a set of diseases, such as asthma, diabetes, hypertension, malaria and tuberculosis. The knowledge of experts–doctors is integrated with the fuzzy decision-making process. In Ref. [22], the authors used a fuzzy inference system (Type 2) for the diagnosis of diabetes mellitus. Sometimes the design of a knowledge base for a fuzzy system is tedious and time-consuming. To automate this process, the knowledge base of the system is often designed from the statistical data. For this aim, the integration of neural networks or evolutionary computation algorithms with statistics is considered. This approach simplifies the design process of the knowledge base of the system. In Ref. [23], the authors used the ANFIS model that integrates fuzzy logic and neural networks for the diagnosis of COVID-19. In Ref. [24], the authors used an evolutionary programming algorithm for the design of a fuzzy model for DNA coding. In Ref. [25], the authors used a Pythagorean fuzzy algorithm for diagnosis problems.

Sometimes, the constructed type-1 fuzzy system cannot handle uncertainties when there are ambiguities in expressing fuzzy terms and when the information used in the knowledge base carries uncertainties. In these conditions, one of the effective ways is the use of type-2 fuzzy logic in expressing the knowledge base [26]. The type-2 fuzzy logic was improved by Mendel and his coauthors [26]. Type-2 fuzzy logic provides a good framework for handling high-order uncertainties due to its three-dimensional membership function. A set of research studies has been performed using type-2 fuzzy logic. These are related to the solution of engineering problems [26,27,28,29] and economic problems [30]. Type-2 fuzzy logic is used for solving medical diagnosis problems—these are the design of a diagnostic system for diabetes [31], and for controlling the glucose level of blood [32]. Neural networks and evolutionary computation algorithms that can be effectively used for designing the type-2 fuzzy system’s knowledge base.

The contributions of the paper include:

- The T2-FNN system based on the integration of type-2 fuzzy system and neural networks is proposed for the detection of fetal health status.
- The structure of type-2 fuzzy neural networks (T2-FNN) is proposed and the design algorithm of T2-FNN is presented.
- The presented system is implemented for the detection of the fetal health status of pregnant women. The simulation of the proposed system is implemented using statistical data.
- Based on the input–output relationship, novel data preprocessing algorithm is designed and tested on the dataset.
- The proposed T2-FNN system shows better accuracy performance in comparison with other models, which enhanced the effectiveness of the fetal health status detection system.

The paper is organized as follows: In Section 2, the prediction of fetal health is performed utilizing the type-2 fuzzy system’s learning capabilities and an exploratory examination of the CTG data. In Section 3, experimental findings and algorithm validation using various evaluation criteria such as the accuracy and efficiency of the proposed model are presented. Finally, the conclusion and potential for future improvement of this research work are presented in Section 4.

## 3. T2-FNN for Detection of Fetal Health Status

A fetal health state is characterized by 21 input symptoms. Laboratory analysis and measurements were applied for the determination of these parameters. Based on the possible values of these parameters, three output diagnoses of fetal healthy states were determined. These are Normal, Suspected and Pathological. Using the number of input and output symptoms, the structure of the T2-FNN system was determined. Here, the basic problem is the determination of the association between input and output variables. We used a type-2 fuzzy rule-based system for the description of these associations. TSK type-2 fuzzy rules were used for the descriptions of these rules. The rules are presented as follows:

(1) $$\mathrm{If}\;x_{1}\;\mathrm{is}\;{\tilde{C}}_{1j}\;\mathrm{and},\ldots ,\;\mathrm{and}\;x_{m}\;\mathrm{is}\;{\tilde{C}}_{mj},\;\mathrm{Then}\;{y_{1}=\sum_{i=1}^{m}v_{i1}x}_{i}\;\mathrm{and},\ldots ,\;\mathrm{and}\;{y_{p}=\sum_{i=1}^{m}v_{ip}x}_{i},$$

where ${\tilde{C}}_{ij}$ values are the interval of type-2 fuzzy values, $v_{ik}$ values are weight coefficients between the rule layer and output layer, *x_i_* and *y_k_* are input and output variables of the system, respectively, *i* = 1, …, *m*, *j* = 1, …, *n*, *k* = 1, …, *p*. *m*, *n* and *p* are the number of inputs, rules, and outputs, correspondingly.

**Figure 1:**
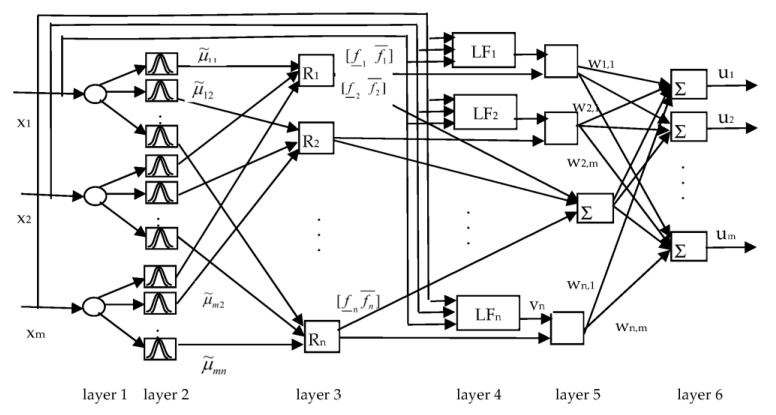
Structure of T2-FNN for detection of fetal healthy status.

Based on fuzzy if–then rules, the structure of T2-FNN is presented (Figure 1). The network is multi-input multi-output that uses a set of inputs and produces a set of outputs. The network includes n fuzzy rules and n local functions (LF).

**Figure 2:**
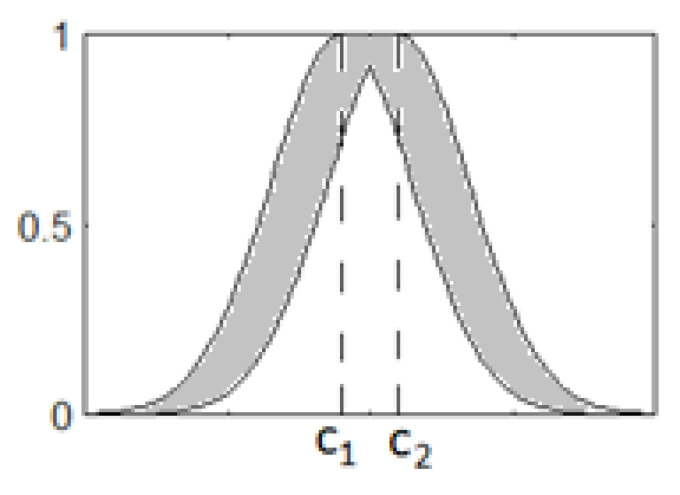
Interval type-2 membership function with uncertain mean.

The ${\tilde{C}}_{ij}$ type-2 values are represented using Gaussians. Here, the uncertainty may be assigned to the mean and width of the Gaussians. We used type-2 fuzzy values with an uncertain mean (Figure 2). The membership function is determined as

(2) $${\tilde{\mu }}_{j}\left(x_{i}\right)=e^{-\frac{{\left(x_{i}-{\tilde{c}}_{ij}\right)}^{2}}{\sigma_{ij}^{2}}}.$$

Here, ${\tilde{c}}_{ij}\in \left[c_{ij}^{1},c_{ij}^{2}\right]$ are the center of the Gaussian membership function (MF) represented by the interval of type-2 fuzzy sets, $\sigma_{ij}$ values are the width of the MF. Each value of the interval of the type-2 MF is presented by a lower ${\underline{\mu }}_{j}(x_{i})$ and upper ${\overset{-}{\mu }}_{j}(x_{i})$ MFs.

(3) $${\tilde{\mu }}_{j}\left(x_{i}\right)=\left[{\underline{\mu }}_{j}\left(x_{i}\right),{\bar{\mu }}_{j}\left(x_{i}\right)\right].$$

Figure 1 depicts the structure of the T2-FNN system used for the detection of fetal health status. When input signals are entered into the network input layer, using Formula (2), lower and upper levels of MPs are determined for each rule. Using the t-norm min (Π) operation, the firing strength of each rule is determined. This is performed using the following equations:

(4) $${\underline{f}}_{j}=\prod_{i=1}^{m}{\underline{\mu }}_{j}\left(x_{i}\right);\;{\bar{f}}_{j}=\prod_{i=1}^{m}{\bar{\mu }}_{j}\left(x_{i}\right)$$

After finding the firing strengths of the rules, type reduction and defuzzification are applied to find the output of the T2-FNN. Firing strengths are used to determine the output of the T2-FNN. The inference engine formulas presented in [27,29,30] are applied to calculate the crisp system’s output.

(5) $$y_{j}=\sum_{i=1}^{m}x_{i}v_{ij},$$

(6) $$u_{k}=p\frac{\sum_{j=1}^{n}{\underline{f}}_{j}y_{j}w_{jk}}{\sum_{j=1}^{n}{\underline{f}}_{j}}+q\frac{\sum_{j=1}^{n}{\bar{f}}_{j}y_{j}w_{jk}}{\sum_{j=1}^{n}{\bar{f}}_{j}},i=1,\;\ldots ,\;m,\;j=1,\;\ldots ,\;n,\;k=1,\;\ldots ,\;p.$$

Here, *x_i_* and *y_j_* are the input and output variables of the system, *w_jk_* are weight coefficients, *p* and *q* are coefficients used to update the lower and upper parts of output signal *u_k_*.

The training of the T2-FNN is carried out by comparing the current output signal with the training output signal. The following formula determines the Euclidean distance between the current output signal and the training output signal:

(7) $$E=\sum_{k=1}^{p}{\left(u_{k}^{d}-u_{k}\right)}^{2},$$

where $u_{k}^{d}\;\mathrm{and}\;u_{k}$ are desired and current outputs of the T2-FNN. Using the error function, the gradient descent algorithm is applied for training the T2-FNN system. As a result of the training, the unknown coefficients of the network $c_{ij}^{1},c_{ij}^{2},\sigma_{ij},$  *v_ij_* and *w_jk_* are determined.

The designed algorithm of the T2-FNN system for the detection of fetal health status is presented in Algorithm 1.
**Algorithm 1** The design stages of T2-FNN systemInput datasets X. Randomly generate initial values of the ***c*1**, ***c*2**, ***o*** parameters of the antecedent part and ***w*1** and ***w*2** parameters of the consequent part of T2FNN. Set maximum epoch number *max_epoch*, learning rate, momentum rate, number of inputs, hidden and output neurons and learning coefficients. Set fold number *K* = 10, current epoch number equal *epoch* = 1. Set initial values of the parameters *p* = 0.5 and *q* = 0.5. **While** *epch <*= *maximum_epoch*, **do**  Partition datasets into *K* groups.  **For each fold, do**    Determine training and validation (testing) samples.    Determine input–output training pairs.   **For** each input–output training pairs, **do**      For each input data using Formulas (2)–(6), determine the output of the T2-FNN. Using *U_T2FNN_* current and *U^d^* desired output signals, calculate the output error *e*(*t*) = *U^d^* − *U_T2FNN_* of the network.      **If**
*abs*(*e*(*t*) > Δ), **Then**        Update ***c*1, *c*2, *o*, *w*1** and ***w*2** parameters of the network. Here (Δ is an acceptable small value).      **endIf**    **endFor**    Calculate the mean of square errors (SSE) and root mean square errors (RMSE) for the training data.    According to gradient of error, adjust the learning rate    Determine input–output validation (testing) pairs    **For** each input–output validation pairs, **do**      For each input validation data using Formulas (2)–(6), determine the output of the T2-FNN.      Calculate the output error *e*(*t*) = *U^d^* − *U_T2-FNN_* of the network.    **endFor**    Determine MSE and RMSE values for validation data (evaluation).  **endFor**  If the current RMSE value for training data is less than the one obtained in the previous epoch, save the T2FNN parameters in the file. **endWhile**.

The algorithm is employed for the design of the T2-FNN-based fetal health status detection system.

## 4. Simulation of the T2-FNN System for Determination of the Fetal Status

### 4.1. Data Preprocessing

The design of the T2-FNN system for finding fetal health status is considered. We used fetal status dataset from the Machine Learning Repository of California Irvine University, which consists of 2126 fetal cardiotocography pieces of data of pregnant women. The dataset is also available on the Kaggle Community https://www.kaggle.com/datasets/andrewmvd/fetal-health-classification, accessed on 25 January 2023. The dataset contains 21 input parameters that include the measurements of FHR and UC from the records of CTG. These are baseline heart rate, number of fetal movements, number of accelerations per second, etc. The descriptions of input parameters are presented in Table 1. Analysis has shown that the data are imbalanced, that is, each class has a different number of data samples. The data have been expertly categorized as Normal, Suspect, and Pathological. The three classes represented in the dataset are Pathological (P), Suspected (S) and Normal (N), respectively. The fragment of input data is presented in Table 2.

The dataset includes the measurements of FHR and UC. Each record in Table 2 includes 21 features, 11 of which were obtained from the electronic sensors, and the other 10 features were extracted using these 11 features. Statistical measurements of datasets are presented in Table 3. Here, for each data item, the values of mean, standard deviation and the *min* and *max* values of each attribute are determined. The statistical measurements have been conducted in order to demonstrate the deviations of the input parameters and their effect on the output results. If we consider the mean values of the input variables and their corresponding standard deviation, we see that the values of some variables are varied in a relatively big interval. The difference in the values of input parameters indicates that this can significantly change the output result.

**Figure 3:**
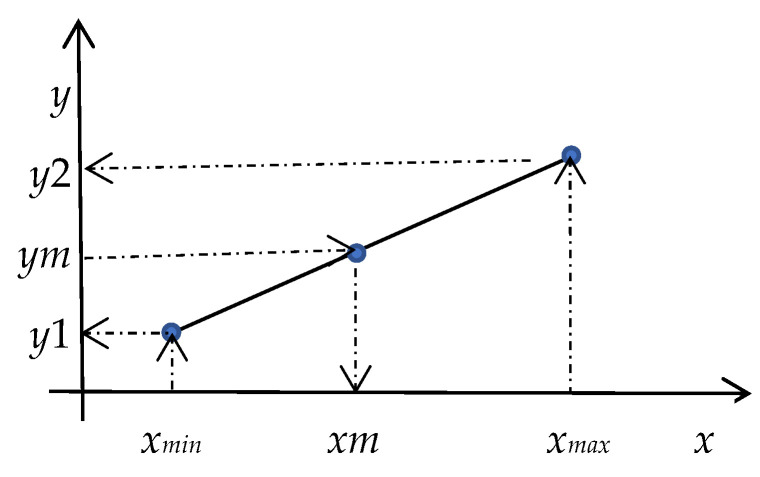
Linear relationship between input and output data.

Sometimes input variables in the dataset have missing values. The authors of paper [33] used the mean values of corresponding features to replace missing values in the dataset. However, this algorithm does not take into account the relationship between input and output variables. Therefore, the constructed model does adequately describe the input–output relationship of the model. To deal with the problem, we have designed an algorithm based on the input–output relationships and linear interpolation. In the designing of the algorithm, we assume that the input–output relationship is either increasing linearly or decreasing linearly. For simplicity, let us consider a single-input single-output relationship. Figure 3 depicts the increasing linear relationship. We assume that the *y*1 output value is corresponding to the *x_min_* input value and the *y*2 output value is corresponding to the *x_max_* value. According to the number of classes, we divided [*y*1, *y*2] and [*xmin*, *xmax*] intervals into subintervals. To determine the missing *xm* value, we use the output *ym* value in the dataset. The basic steps of the algorithm are presented below.

1.  Find a number of output classes *nc*.
2.  Read the values of input–output variables from the dataset.
3.  Find the missing input variable *xm* and determine its minimum *x_min_* = *min*(*xm*) and maximum *x_max_* = *max*(*xm*) values.
4.  Determine minimum *y*1 and maximum *y*2 values of output classes corresponding to missing input *xm* variables.
5.  Determine the steps for the missing input and corresponding output variables using formulas $\Delta x=\frac{x_{max}-x_{min}}{nc-1}$ and $\Delta y=\frac{y2-y1}{nc-1}$.
6.  Using minimum and maximum values, determine cluster centers for the missing input and corresponding output variables using formulas
  $x_{c}^{k}=x_{min}+(k-1)\Delta x$ and $y_{c}^{k}=y1+(k-1)\Delta y$. Here, *k* = *1*, *…*, *nc*, *c* = 1, *…*, *C*. *nc* is the number of output classes, *C* is the number of input variables.
7.  Save the $x_{c}^{k}$ and $y_{c}^{k}$.
8.  Repeat Steps 3–7 for all missing variables.
9.  Read the dataset, find the row that has missing input value xm in the dataset and fix the corresponding output class $y_{c}^{k}$ of this row.
10. Use $y_{c}^{k}$ (Step 9) and the stored input–output data (Step 7) to find the corresponding input $x_{c}^{k}$ data.
11. Replace the missing value of *xm* in the dataset with the value of $x_{c}^{k}$ determined in Step 10.
12. Repeat Steps 9–11 for all missing values.

Using input and output statistical datasets, the design of T2-FNN is performed. We measure recall, precision, F1-score and accuracy to evaluate the performance of the designed T2-FNN model. For this purpose, we determine the number of false negative (*FN*), false positive (*FP*), true negative (*TN*) and true positive (*TP*) predictions produced by the model. Using these variables, the values of recall, precision, F1-score and accuracy are evaluated.

(8) $$Accuracy=\frac{TP+TN}{TP+TN+FP+FN},$$

(9) $$Precision=\frac{TP}{TP+FP},$$

(10) $$Recall=\frac{TP}{TP+FN},$$

(11) $$f1=\frac{2(precision\times recall)}{Precision+recall}.$$

Accuracy is determined as the ratio between the correct output instances and the total number of output instances of the system. The recall is the ratio between the number of TP instances and the total number of actual positive instances in the dataset. It is a measure of the model’s ability to detect all the positive instances in the dataset. Precision is determined as the ratio between the number of TP instances and the total number of positive instances. F1-score is often used to estimate the model performance when the dataset is imbalanced. A model with a high F1-score makes a good balance between precision and recall. The higher the F1-score (near 1), the better the prediction of the positive instances of the model without infringing precision or recall.

### 4.2. Simulation

**Figure 4:**
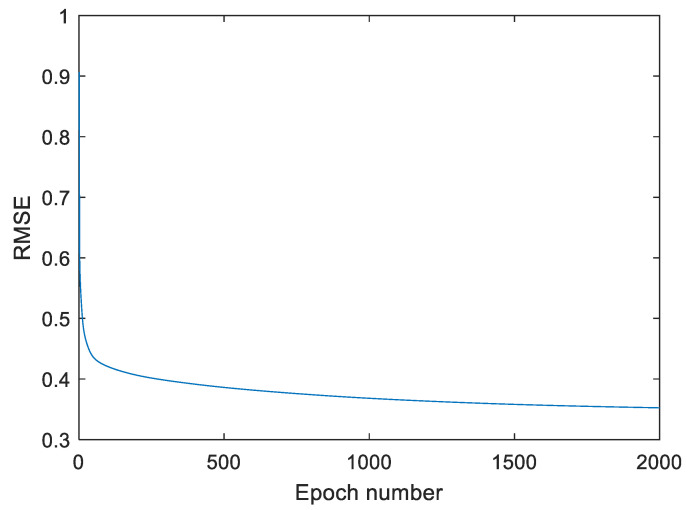
Training Plot.

During simulation, statistical input–output data were applied to train network parameters. In data preprocessing, the input data were scaled within the range of [0, 1]. This process helped to improve the training process of the system. The training is carried out using a gradient descent algorithm used for adjusting unknown coefficients of the T2-FNN system by utilizing a cross-validation approach. Learning is implemented using K-fold cross-validation for 1000 epochs. Cross-validation uses a different portion of data to train and test a model. In the paper, K is taken as 10, that is, 10-fold cross-validation was used for training. Here, the datasets are divided into ten equal groups. Nine groups are used for training and one group for testing which is called validation. The aim is to test the ability of the model to classify (or predict) new data that were not used for training. In this way, we want to see how the model classifies the unknown dataset. In each training iteration, the test group is changed. For example, if, in the first iteration, Group 10 is used for testing (validation) and other groups for training, in the second iteration, Group 9 will be used for validation and the rest for training; in the third iteration, Group 8 will be used for validation, and so on. This process is continued for all data groups. Root mean square error (RMSE) is utilized to test the learning performance of the T2-FNN. During training, the obtained RMSE values for the training and validation data are depicted in Table 4. After finishing the training of the system, we tested the system’s performance using all datasets. The simulation was carried out using the 21, 42, and 63 rules. The learning progress of the T2-FNN with 63 rules is presented in Figure 4. After training, the whole dataset was fed to the T2-FNN input in order to determine the performance of the system. Table 4 depicts the values of RMSE for testing, validation and training data. The table also contains the test results obtained for precision, recall, F1-score and accuracy. As shown in the table, the increase in the number of rules leads to an increase in the performance characteristics of the model. For 63 rules, the RMSE values for training, validation and test data were 0.3188, 0.3233 and 0.3223, respectively. The values of accuracy, precision, recall and F1-score were obtained as 0.966, 0.952, 0.952 and 0.952, correspondingly.

We tested the data preprocessing algorithm and simulated the health status problem. For this purpose, we randomly erased 150 input pieces of data from the dataset. The designed data preprocessing algorithm was used to determine the actual values of these input values. After recovering the new values of missing data, we trained and tested the system using 21 fuzzy rules. We obtained RMSE values for training, validation and testing as 0.39764, 0.41012 and 0.40785, respectively. These are the averaged results of 10 simulations. As shown, the obtained results with and without data preprocessing are nearly the same. The results indicate the suitability of using the designed preprocessing algorithm.

We also performed simulations of the fetal health status detection system using several machine learning techniques. For simulation purposes, we used logistic regression, Gaussian Naïve Bayes, SVC (support vector classification), RBF SVC (radial basis function kernel SVC), ANN (artificial neural networks), CART (classification and regression trees), Random Forest, RNN (Recurrent Neural Networks) and CatBoost (Category and Boosting) algorithms. The values of precision, recall, F1-score and accuracy were used to measure the performances of the models. Table 5 shows the results obtained from the simulations of the different machine learning models. As shown, the results obtained from the T2-FNN model are better than the ones obtained from other models.

In the next stage, the results of simulations of the T2-FNN system were compared with the results of the other systems used for the determination of the fetal health status of pregnant women. We analyzed the existing research works that used the same fetal dataset. This comparison was carried out in order to prove the suitability of the designed system. Table 6 presents the comparative results of various models. As shown in Table 6, T2-FNN model with 21, 42 and 63 rules have better performances than others. The provided results indicate the suitability and efficiency of using T2-FNN for the determination of fetal health status.

## 5. Conclusions

The integration of type-2 fuzzy sets and neural network structure is proposed for the design of T2-FNN to determine fetal health status. The structure of T2-FNN is presented, and the design of the system is carried out. A cross-validation technique with the gradient algorithm is used for the construction of the system. The constructed T2-FNN is tested using fetal datasets. Different numbers of rules are employed for the system design. It is found that the increase in the number of rules leads to an increase in the performance of the system. The values of precision, recall, F1-score and accuracy are determined as 95%, 95%, 95% and 96.6%, respectively. The performance of the T2-FNN system is compared with the performances of other systems used for the determination of fetal health status. The obtained comparison indicates the suitability of using T2-FNN in the determination of fetal health status.

## Figures and Tables

**Table 1:** Input symptoms.

Attribute	Description
BV	FHR baseline value—beats per min
AC	No. of accelerations per sec
FM	No. of fetal movement
UC	No. of uterine contractions per sec
LD	No. of light decelerations per sec
SD	No. of severe decelerations per sec
PD	No. of prolonged decelerations per sec
ASTV	Percentage of time with abnormal short-term variability
MSTV	Mean Value of Short-Term Variability
ALTV	Percentage of Time with Abnormal Long-Term Variability
MLTV	Mean Value of Long-Term Variability
HW	Histogram Width (width of FHR histogram)
HMax	Histogram Max (maximum of FHR histogram)
Hmin	Histogram Min (minimum of FHR histogram)
NP	Number of Histogram Peaks
NZ	Number of Histogram Zeroes
HMo	Histogram Mode
HMe	Histogram Mean
HMed	Histogram Median
HV	Histogram Variance
HT	Histogram Tendency
NSP	Fetal Health (Fetal state class code, N = normal, S = Suspected, P = Pathological)

**Table 2:** Fragment of input data.

151.0	…	64.0	1.9	9.0	27.6	130.0	56.0	186.0	2.0	0.0	150.0	148.0	151.0	9.0	1.0	2.0
150.0	…	64.0	2.0	8.0	29.5	130.0	56.0	186.0	5.0	0.0	150.0	148.0	151.0	10.0	1.0	2.0
131.0	…	28.0	1.4	0.0	12.9	66.0	88.0	154.0	5.0	0.0	135.0	134.0	137.0	7.0	1.0	1.0
131.0	…	28.0	1.5	0.0	5.4	87.0	71.0	158.0	2.0	0.0	141.0	137.0	141.0	10.0	1.0	1.0
130.0	…	21.0	2.3	0.0	7.9	107.0	67.0	174.0	7.0	0.0	143.0	125.0	135.0	76.0	0.0	1.0
130.0	…	19.0	2.3	0.0	8.7	107.0	67.0	174.0	3.0	0.0	134.0	127.0	133.0	43.0	0.0	1.0
130.0	…	24.0	2.1	0.0	10.9	125.0	53.0	178.0	5.0	0.0	143.0	128.0	138.0	70.0	1.0	1.0

**Table 3:** Statistical measurements.

Input Variables	Mean	Std	Min	Max
baseline value	133.303857	9.840844	106.0	160.0
accelerations	0.003178	0.003866	0.0	0.019
fetal_movement	0.009481	0.046666	0.0	0.481
uterine_contractions	0.004366	0.002946	0.0	0.015
light_decelerations	0.001889	0.002960	0.0	0.015
severe_decelerations	0.000003	0.000057	0.0	0.001
prolongued_decelerations	0.000159	0.000590	0.0	0.005
abnormal_short_term_variability	46.990122	17.192814	12.0	87.000
mean_value_of_short_term_variability	1.332785	0.883241	0.2	7.000
percentage_of_time_with_abnormal_long_term_variability	9.846660	18.396880	0.0	91.000
mean_value_of_long_term_variability	8.187629	5.628247	0.0	50.700
histogram_width	70.445908	38.955693	3.0	180.000
histogram_min	93.579492	29.560212	50.0	159.000
histogram_max	164.025400	17.944183	122.0	238.000
histogram_number_of_peaks	4.068203	2.949386	0.0	18.000
histogram_number_of_zeroes	0.323612	0.706059	0.0	10.000
histogram_mode	137.452023	16.381289	60.0	187.000
histogram_mean	134.610536	15.593596	73.0	182.000
histogram_median	138.090310	14.466589	77.0	186.000
histogram_variance	18.80809	28.977636	0.0	269.000
histogram_tendency	0.320320	0.610829	−1.0	1.000
fetal_health	1.304327	0.614377	1.0	3.000

**Table 4:** Simulation results.

No	Training Error	Validation Error	Test Error	Accuracy	Precision	Recall	F1 Score
21	0.393879	0.409894	0.407634	0.936	0.9036	0.9038	0.9038
42	0.351097	0.361881	0.361453	0.958	0.9375	0.9375	0.9375
63	0.318810	0.323347	0.322312	0.966	0.9518	0.9518	0.9518

**Table 5:** Comparison with machine learning algorithms.

Method	Accuracy	Precision	Recall	F-1 Score
Logistic Regression	0.89	0.87	0.88	0.87
Gaussian Naive Bayes	0.79	0.86	0.80	0.82
SVC	0.88	0.88	0.88	0.88
RBF SVC	0.90	0.91	0.91	0.91
ANN	0.91	0.91	0.92	0.91
CART	0.93	0.93	0.93	0.93
Random Forest	0.94	0.94	0.94	0.94
CatBoost	0.93	0.94	0.94	0.94
RNN	0.92	0.92	0.92	0.91
T2-FNN (21 rules)	0.936	0.904	0.904	0.904
T2-FNN (42 rules)	0.958	0.94	0.94	0.94
T2-FNN (63 rules)	0.966	0.95	0.95	0.95

**Table 6:** Comparative results.

The Research Works	Methodology	Accuracy
Sharma P [12]	Decision Tree	92.63
Sharma P [12]	K-NN	92.78
Sharma P [12]	Random Forest	93.57
Agrawal [14]	Decision Tree	91.54
Agrawal [14]	SVM	92.39
Agrawal [14]	Naïve Bayes	85.572
Mehbodniya [15]	K-NN	91.23
Mehbodniya [15]	SVM	93
Mehbodniya [15]	MLP	92.53
Mehbodniya [15]	Random Forest	94.5
Current Research	T2-FNN (21 rules)	93.6
Current Research	T2-FNN (42 rules)	95.8
Current Research	T2-FNN (63 rules)	96.66

## Data Availability

The dataset explored in the research can be found at https://www.kaggle.com/andrewmvd/fetal-health-classification, accessed on 25 January 2023. The dataset is made up of 2126 CTG data.
